# Design optimization of stent and its dilatation balloon using kriging surrogate model

**DOI:** 10.1186/s12938-016-0307-6

**Published:** 2017-01-11

**Authors:** Hongxia Li, Tao Liu, Minjie Wang, Danyang Zhao, Aike Qiao, Xue Wang, Junfeng Gu, Zheng Li, Bao Zhu

**Affiliations:** 1 School of Mechanical Engineering, Dalian University of Technology, Dalian, 116023 Liaoning China; 2 College of Life Science and Bioengineering, Beijing University of Technology, Beijing, 100124 China; 3 Mechanical Engineering and Material Science Department, University of Pittsburgh, 4200 Fifth Avenue, Pittsburgh, PA 15260 USA; 4 Department of Engineering Mechanics, State Key Laboratory of Structural Analysis for Industrial Equipment, Dalian University of Technology, Dalian, 116023 Liaoning China; 5 School of Materials Science and Engineering, Dalian University of Technology, No. 2, Linggong Road, Dalian, 116024 Liaoning China

**Keywords:** Stent, Fatigue life, Dogboning effect, Finite element method, Kriging surrogate model, Design optimization

## Abstract

**Background:**

Although stents have great success of treating cardiovascular disease, it actually undermined by the in-stent restenosis and their long-term fatigue failure. The geometry of stent affects its service performance and ultimately affects its fatigue life. Besides, improper length of balloon leads to transient mechanical injury to the vessel wall and in-stent restenosis. Conventional optimization method of stent and its dilatation balloon by comparing several designs and choosing the best one as the optimal design cannot find the global optimal design in the design space. In this study, an adaptive optimization method based on Kriging surrogate model was proposed to optimize the structure of stent and the length of stent dilatation balloon so as to prolong stent service life and improve the performance of stent.

**Methods:**

A finite element simulation based optimization method combing with Kriging surrogate model is proposed to optimize geometries of stent and length of stent dilatation balloon step by step. Kriging surrogate model coupled with design of experiment method is employed to construct the approximate functional relationship between optimization objectives and design variables. Modified rectangular grid is used to select initial training samples in the design space. Expected improvement function is used to balance the local and global searches to find the global optimal result. Finite element method is adopted to simulate the free expansion of balloon-expandable stent and the expansion of stent in stenotic artery. The well-known Goodman diagram was used for the fatigue life prediction of stent, while dogboning effect was used for stent expansion performance measurement. As the real design cases, diamond-shaped stent and sv-shaped stent were studied to demonstrate how the proposed method can be harnessed to design and refine stent fatigue life and expansion performance computationally.

**Results:**

The fatigue life and expansion performance of both the diamond-shaped stent and sv-shaped stent are designed and refined, respectively. (a) diamond-shaped stent: The shortest distance from the data points to the failure line in the Goodman diagram was increased by 22.39%, which indicated a safer service performance of the optimal stent. The dogboning effect was almost completely eliminated, which implies more uniform expansion of stent along its length. Simultaneously, radial elastic recoil (RR) at the proximal and distal ends was reduced by 40.98 and 35% respectively and foreshortening (FS) was also decreased by 1.75%. (b) sv-shaped stent: The shortest distance from the data point to the failure line in the Goodman diagram was increased by 15.91%. The dogboning effect was also completely eliminated, RR at the proximal and distal ends was reduced by 82.70 and 97.13%, respectively, and the FS was decreased by 16.81%. Numerical results showed that the fatigue life of both stents was refined and the comprehensive expansion performance of them was improved.

**Conclusions:**

This article presents an adaptive optimization method based on the Kriging surrogate model to optimize the structure of stents and the length of their dilatation balloon to prolong stents fatigue life and decreases the dogboning effect of stents during expansion process. Numerical results show that the adaptive optimization method based on Kriging surrogate model can effectively optimize the design of stents and the dilatation balloon. Further investigations containing more design goals and more effective multidisciplinary design optimization method are warranted.

## Background

Cardiovascular and cerebrovascular diseases pose a great threat to human beings. Since 1990s, minimally invasive procedures have been introduced to deal with vascular diseases such as percutaneous transluminal coronary angioplasty (PTCA) with stent, which has been widely used in clinical treatment and become one of the most effective therapies to vascular diseases. Compared to drugs and traditional surgeries, this newly developed minimally invasive treatment enjoys a lot of advantages such as being effective and efficient, being relatively easy to perform, causing only minor trauma to patients, ensuring a low infection rate and leading to relatively low cost [1]. However, the development and clinical application of this technology has been impeded by many factors including long-term safety problem of stents, in-stent restenosis (ISR) due to mechanical injury caused by the stent to vascular wall and inflammatory response of vessel wall against struts. Obviously, stent long-term safety is related to its fatigue life in-service loading and non-uniform stent expansion will cause mechanical damage to the artery wall which has a significant impact on thrombosis and hyperplasia development [2].

As for percutaneous transluminal coronary angioplasty, stent is placed into the stenosis segment of vessel to provide a mechanical support and then the balloon and catheter are removed away. The stent remains in vessel to support vascular wall to ensure smooth blood flow. It also means that the stent would suffer pulsating load all the time in vessel. According to FDA [3], the service life of stent shall be no less than 10 years which means that it should withstand at least 380 million pulsation cycles. FDA also recommends several methods such as Goodman diagrams to test lifetime of stent. Currently, limited by minute structure of stent and vessel as well as the complexity of hemodynamics in stent, researchers often adopt experiment to study fatigue life of stent. However, it often takes 2–3 months to perform the accelerated life test to analyze stent’s fatigue life [4]. Against such a background, it is practically meaningful to explore how to use numerical simulation method to analyze stent’s fatigue life and then optimize geometries of stent based on numerical simulation so as to prolong the service life of stent.

The expanding of stent is not only affected by its geometries but also influenced by the balloon length. Mortier et al. [5] highlighted that the length of balloon is likely to be related to the expanding of stent’s distal ends. It means that under the influence of balloon length the stent may finally take up the shape of a spindle because the distal ends fail to expand enough or it may take the shape of a dogbone because the distal ends expand too much. Such a non-uniform stent expansion may cause mechanical injury to vessel wall and thus leading to in-stent restenosis. Therefore, it is practically meaningful to find out the proper length of balloon so as to ensure that the stent achieves uniform expansion along its length and to reduce mechanical injury to vessel wall.

Therefore, it is important in stenting to predict and optimize the fatigue life and expansion performance before manufacturing the stent and its dilatation balloon. However, it is hard for traditional methods such as experiment and clinical tests to find the optimal result in stent optimization since the functional relationship between design objectives and variables is nonlinear, complex and implicit. Currently, the common method to optimize stent is to compare several stent designs and choose the best one among them. For example, Migliavacca et al. [6], De Beule et al. [7] and Wang et al. [8] compared the expanding performance of the same type of stent with different geometrics and gave suggestions on the design of stent. This method is relatively easy to use but the optimal stent is actually the relatively better one among a couple of options rather than the real optimal result in the design space. What’s more, since the dilatation of balloon-expandable stent entails highly nonlinear problems such as large deformation, contact and elasto-plasticity [9–12], it is difficult to perform optimization by adopting finite element method. As a matter of fact, comparing and analyzing a large amount of geometries of stent and its balloon are time-consuming and costly.

Fortunately, surrogate model can solve such tricky problems. It is the use of a black box model to create an approximate functional relationship between design objectives and variables, thereby replacing complex engineering computation so as to greatly reduce computational cost. Timmins et al. [13] adopted Lagrange interpolating polynomials (LIPs) to optimize the stent; Shen et al. [14] improved stent’s resistance against compression and decreased internal pressure in expanding stent by employing the artificial neural networks (ANN). Li et al. [15, 16] proposed an adaptive optimization method based on Kriging surrogate model to optimize stent structure to eliminate the dogboning phenomenon during stent expansion process and optimize stent coating to prolong the effective period of drug release. Kriging surrogate model, a semi-parameter interpolation technique, is more precise and flexible compared to Lagrange interpolating polynomials and ANN, and thus widely used in multi-disciplinary design optimization (MDO).

In the present paper, both the expansion performance of stent and the fatigue life of stent in-service loading were studied. The stent geometries and its dilatation balloon were optimized step by step to improve stent fatigue life and expansion performance. The Kriging model was used to build the relationship between stent fatigue life and stent geometries and the relationship between stent dogboning ratio and length of balloon, respectively, thereby replacing the expensive FEM reanalysis of the fatigue life and dogboning ratio during the optimization. The optimization iterations are based on the approximate relationships for reducing the high computational cost. A ‘space-filing’ sampling strategy conceptualized as a rectangular grid was used to generate the initial training sample points. In the adaptive optimization process, EI function was adopted to balance local and global searches and tends to find the global optimal design, even with a small sample size. In the present study, an adaptive optimization method was proposed for stent and its dilatation balloon optimization to prolong stent fatigue life and improve its expansion performance, which is hard and time-consuming to find the optimal design either by experiment or clinic test. As the real design cases, two typical and representative vascular stents named diamond-shaped stent and sv-shaped stent were studied to demonstrate how the proposed method can be harnessed to design and refine stent fatigue life and expansion performance computationally. The numerical results and design optimization method can provide a reference for the design of stent and its dilatation balloon.

## Methods

### Finite element analysis

**Fig. 1:**
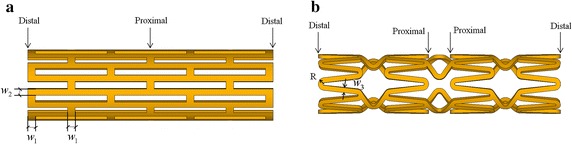
Geometries and design variables of optimization to improve stent fatigue life. **a** Palmaz-Schatz (*diamond*-*shaped*) stent platform, **b** *sv*-*shaped* stent platform

A typical diamond-shaped stent and sv-shaped stent were optimized in this study (as shown in Fig. 1). Finite element method (FEM) is used to simulate expansion process of stent. Geometries of stent and balloon come from relevant literatures [17, 18]. Data on material properties of vascular tissue and balloon derives from relevant literatures [17, 19, 20], as shown in Table 1. The diamond-shaped stent with 8.68 mm in length and 2.54 mm in outside diameter, and sv-shaped stent with 6.05 mm in length and 1.5 mm in outside diameter are fixed to be equal to a bilinear isotropic elastic model; the vascular tissue is taken as incompressible linear elastic material; the balloon is assumed to be hyper-elastic material.

1. FEM simulation for the stent fatigue life prediction (FLP): Numerical simulation of the stent deployment derives from relevant literatures [20], which conducted in three steps: first, deployment of stent inside the stenotic artery by imposing a radial displacement to the balloon. Then, stent recoil upon balloon deflation by removing the deployment radial displacement to the balloon. Finally, cardiac cycle of pulsating load by applying a diastolic/systolic blood pressure to the artery.

**Fig. 2:**
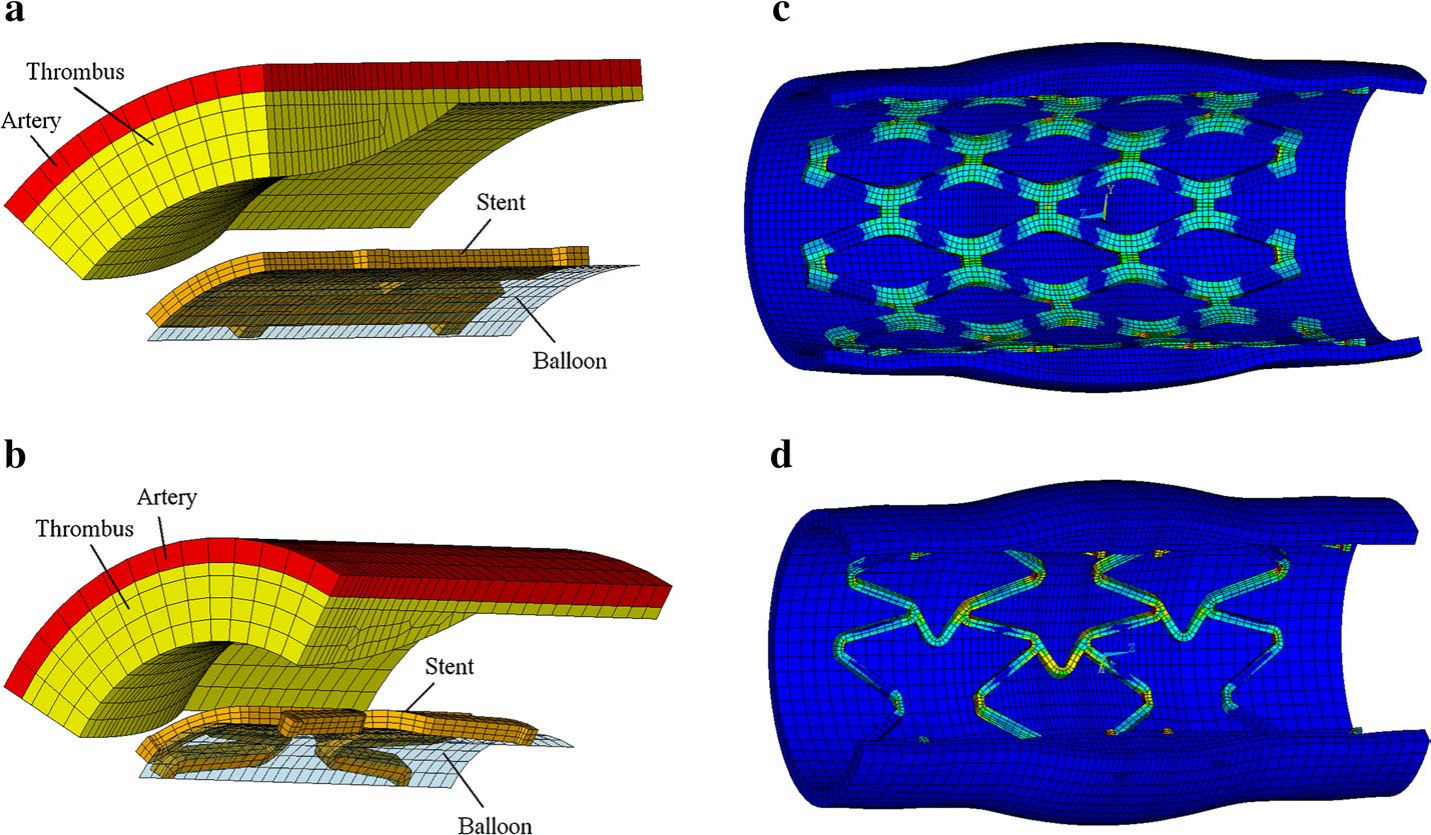
Finite element model of stent expansion in stenotic artery based on *diamond*-*shaped* and *sv*-*shaped* artery platforms. **a** FEM model of diamond-shaped stent expansion in stenotic artery, **b** FEM model of *sv*-*shaped* stent expansion in stenotic artery, **c** *diamond*-*shaped* stent expansion in stenotic artery, **d** *sv*-*shaped* stent expansion in stenotic artery

As the pattern repeats itself symmetrically, 1/16 of the model of diamond-shaped stent (1/8 in circumferential direction and 1/2 in axis direction) and 1/8 of the model of sv-shaped stent (1/4 in circumferential direction and 1/2 in axis direction) were modeled (as shown in Fig. 2). Symmetry boundary condition is applied in the axial direction and rigid body displacement constraint is applied in the circumferential direction.

Fatigue life prediction of the stent was conducted using the well-known Goodman diagram (GD) [3] which represents a plot of the stress amplitude *σ*_*a*_ versus mean stress *σ*_*m*_. The failure line is defined by Goodman equation:

1 $$\frac{{\sigma_{a} }}{{\sigma_{N} }} + \frac{{\sigma_{m} }}{{\sigma_{UTS} }} = 1$$

where *σ*_*a*_ is the amplitude of the applied cyclic stress (*σ*_*a*_ = |*σ*_*systolic*_ − *σ*_*diastolic*_|/2, *σ*_*m*_ is the mean of the applied stress (*σ*_*m*_ = (*σ*_*systolic*_ + *σ*_*diastolic*_)/2), *σ*_*N*_ is the endurance limit, *σ*_*UTS*_is the ultimate tensile strength. *σ*_*N*_ and *σ*_*UTS*_ are the material properties determined by experimental high cycle fatigue tests. In this study, the material properties of the stents are as given in relevant literature [20].

**Fig. 3:**
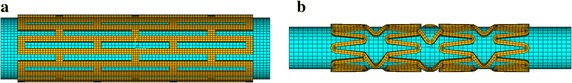
Numerical simulation models of balloon-stent free expansion. **a** *diamond*-*shaped* stent platform, **b** *sv*-*shaped* stent platform

(2) FEM simulation for stent expansion performance: There are many finite element models (FEM) used to investigate expansion process of stent in the published studies [21–23]. Among them, four common finite element models of stent expansion were used for the design optimization based on Kriging surrogate model to reduce the dogboning effect of stent by Li et al. [24]. From the previous study, the finite element model of stent-balloon expansion with a loading of a time-varying pressure applied to the inner surface of a cylindrical balloon is suitable for design optimization of stent expansion performance using surrogate model combining with FEM,as shown in Fig. 3.

**Fig. 4:**
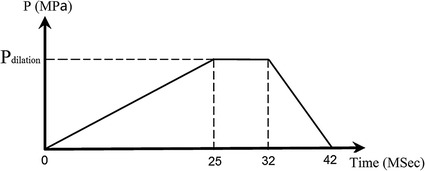
Time-varying pressure including three load phases: 0–25 ms linear loading; 25–32 ms constant loading; 32–42 ms linear unloading [17]

Since the stent has symmetrical structure and boundary conditions, 1/16 of the model of diamond-shaped stent (1/8 in circumferential direction and 1/2 in axis direction) and 1/8 of the model of sv-shaped stent were used to simulate the expanding of stent-balloon system as shown in Fig. 3. Symmetry boundary condition is applied in the axial direction and rigid body displacement constraint is applied in the circumferential direction. Nodes at the distal end of balloon are constrained on rigid-body displacement leaving corresponding nodes at the other end free. The contact between stent and balloon is considered but the friction between them is neglected. Pressure that varies with time (shown in Fig. 4) is applied to inner surface of the balloon [17]. It is noteworthy that the pressure used to dilate stent to its nominal diameter (the diameter of healthy artery) after unloading of balloon varies according to balloon length. Thus binary-search method is employed in the study to find the specific pressure used to dilate stent to its nominal diameter after unloading of balloon.

### Optimization problem

1. Optimization of stent fatigue life: Goodman Diagram is generally employed to predict fatigue life of stent. Data point above or closer to the failure line on the Goodman Diagram indicates that fatigue failure will occur at the zone where the corresponding node located. While, the data point under and far from the failure line indicates a safe service performance. Therefore, the optimization of stent to prolong its fatigue life can be defined as:

2 $$\begin{aligned} \hbox{max} \, D^{shortest} \left( {\mathbf{x}} \right) \hfill \\ {\text{s}} . {\text{t}} . { }{\mathbf{\underset{\raise0.3em\hbox{$\smash{\scriptscriptstyle-}$}}{x} }} \le {\mathbf{x}} \le {\bar{\mathbf{x}}} \hfill \\ \, \frac{{\sigma_{a} }}{{\sigma_{N} }} + \frac{{\sigma_{m} }}{{\sigma_{UTS} }} \le 1 \hfill \\ \end{aligned}$$

where, *D* ^*shortest*^ denotes the shortest distance from the data point to the failure line of stents, **x** is the design variables namely geometries of stent (see Fig. 1), $${\mathbf{\underset{\raise0.3em\hbox{$\smash{\scriptscriptstyle-}$}}{x} }}$$ and $${\bar{\mathbf{x}}}$$ are used to refer to the upper limit and lower limit for design variables respectively. The range of design variables of the diamond-shaped and sv-shaped stents are : 0.22 *mm* ≤ *w*_1_ ≤ 0.34 *mm*, 0.2 *mm* ≤ *w*_2_ ≤ 0.3 *mm*, 0.1 *mm* ≤ *t*_1_ ≤ 0.14 *mm* and 0.08 *mm* ≤ *w*_3_ ≤ 0.12 *mm*, 0.08 *mm* ≤ *R* ≤ 0.15 *mm*, 0.22 *mm* ≤ *t*_2_ ≤ 0.34 *mm*, respectively. *w*_1_, *w*_2_ and *w*_3_ are the struts width of stents shown in Fig. 1. *t*_1_ and *t*_2_ are the thickness of diamond-shaped stent and sv-shaped stent. *R* is the chamfer radius of sv-shaped stent, as shown in Fig. 1.

2. Optimization of stent expansion performance: For balloon-expandable coronary stent, non-uniform expansion along its length often occurs and leads to dogboning effect. It means that the distal ends of stent begin to expand before the proximal part and thus the stent expands into the shape of dogbone. The dogboning ratio can be defined as:

3 $$Dogboning\,Ratio = \frac{{d_{radial}^{distal} - d_{radial}^{proximal} }}{{d_{radial}^{proximal} }}$$

where, *d*_*radial*_^*distal*^ and *d*_*radial*_^*proximal*^ denote the distal and proximal radial displacements of stent respectively.

Dogboning ratio is an important measure of stent expansion. When the dogboning ratio is more than 0, it indicates that the distal ends expand faster than the proximal part and stent takes up the shape of a dogbone. It pushes the struts outward against vessel wall and causes mechanical injury to it. When dogboning ratio is less than 0, it implies that the distal ends expand more slowly than the proximal part and stent takes up the shape of a spindle. Consequently, the struts are pushed inward and may stop the flow of blood. Thus when dogboning ratio tends to zero, the expansion of stent along axial direction is uniform. Moreover, when the stent achieves the maximum expansion at 32 *ms*, it incurs the maximum transient damage to vessel wall. Optimization with the aim of decreasing dogboning ratio during the expanding of stent can be expressed as:

4 $$\begin{aligned} Min \, f({\mathbf{L}}) = \left| {\frac{{d_{radial}^{distal} ({\mathbf{L}}) - d_{radial}^{proximal} ({\mathbf{L}})}}{{d_{radial}^{proximal} ({\mathbf{L}})}}} \right| \hfill \\ S.t \, \underline{{\mathbf{L}}} \le {\mathbf{L}} \le \overline{{\mathbf{L}}} \hfill \\ \end{aligned}$$

where *d*_*radial*_^*distal*^ (**L**) and *d*_*radial*_^*proximal*^ (**L**) denote the distal and proximal radial displacements of stent respectively at 32 ms. *f*(**L**) is the absolute value of dogboning ratio during the expanding of stent, **L** refers to the length of balloon, $${\mathbf{\underset{\raise0.3em\hbox{$\smash{\scriptscriptstyle-}$}}{L} }}$$ and $${\bar{\mathbf{L}}}$$ are the upper limit and lower limit for balloon length. In this study, the design space of L of diamond-shaped stent and sv-shaped stent are 4.6 mm ≤ *L* ≤ 5.1 mm and 6 mm ≤ *L* ≤ 6.5 mm. When the balloon with the length of $${\bar{\mathbf{L}}}$$, the dogboning ratio is larger than 0 and stent takes up the shape of dogbone, while when the balloon with the length of $${\bar{\mathbf{L}}}$$, the dogboning ratio is smaller than 0 and stent takes up the shape of spindle.

Because design objective and design variables in the optimization of stent’s fatigue life are independent from those in the optimization of stent expansion, the optimization process can be divided into two steps namely optimizing stent fatigue life and optimizing stent expansion performance, during which the key geometries of stent and the length of balloon are optimized respectively. There is no iteration involved in the two steps. The stent structure obtained through the optimization of stent’s fatigue life is adopted in the optimization of stent expansion.

**Table 1:** Material properties

Structure	Balloon	Stent	Thrombus	Vessel
Material	Rubber	Stainless steel 304	Calcified thrombus	Calcified vessel
Element type	4-node shell element	8-node solid element	8-node solid element	8-node solid element
Material model	Super-elasticity	Bilinearity, isotropy	Linear, isotropy	Linear, isotropy
Elastic modulus (GPa)	*C*_10_ = 0.10688E − 2, *C*_01_ = 0.0710918E − 2	193	0.00219	0.00175
Poisson’s ratio	0.495	0.3	0.499	0.499
Ultimate tensile strength (GPa)		0.58		
Yield stress(GPa)		0.315		
Endurance limit(GPa)		0.115		

### Optimization algorithm

**Fig. 5:**
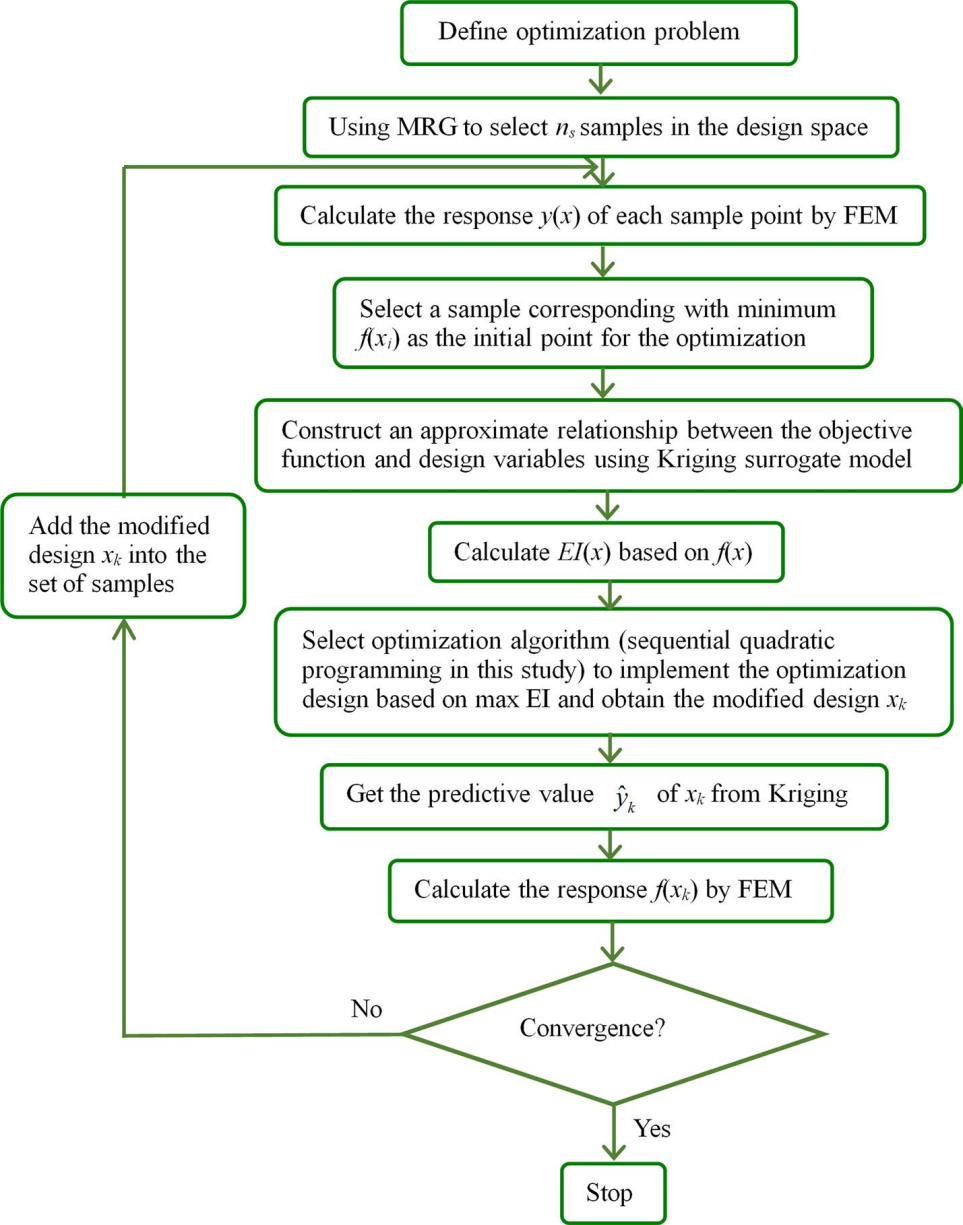
Flow chart of optimization combining with Kriging surrogate modeling

Altered adaptive optimization method based on Kriging surrogate model is employed to minimize the cumulative damage of stent under pulsating load and the absolute value of dogboning ratio during the expanding of stent. Kriging surrogate model [25, 26] coupled with design of experiments (DOE) algorithm [27] is used to create approximate functional relationship between design objective and design variables. The basic idea of Kriging is to predict the value of a function at a given point by computing a weighted average of the known values of the function in the neighborhood of the point. It derives a best linear unbiased estimator, based on assumptions on covariance, makes use of Gauss-Markov theorem to prove independence of the estimate and error, and employs very similar formulae. A new value can be predicted at any new spatial location by combining the Gaussian prior with a Gaussian likelihood function for each of the observed values [28]. As a semi-parametric approach, Kriging model is more flexible in application than interpolation method which involves parametric model and more powerful in making global prediction than semi-parametric model [29]. Altered modified Rectangular Grid (MRG) [15] is adopted to select sample points in the design space of stent’s geometries and in the design space of balloon’s length respectively. It can move some points lying in boundary with the internal design region, which will provide more useful information for the Kriging model, and can ensure that the points have fewer replicated coordinate values. Moreover, it can avoid the case where sample points are spaced close to each other; this may occur when using LHS [15]. Expected improvement (EI) function [27] is adopted to balance the local and global search so as to find the optimal result. The optimization iteration started from a sample point corresponding with minimum *f*(**x**) in training samples, where *f*(**x**) is the optimization objective function, such as cumulative damage of stent and absolute value of dogboning ratio in this study. We modify the Kriging model in each iteration step until the error between Kriging predictive value and FEM simulation falls below a given tolerance. The optimization process stops when the following conditions of convergence are met:

5 $$\begin{aligned} \frac{{EI_{k} }}{{Y_{\hbox{max} } - Y_{\hbox{min} } }} \le \varepsilon_{1} \hfill \\ \left| {f_{k} - \hat{y}_{k} } \right| \le \varepsilon_{2} \hfill \\ \left| {f_{k} - f_{k - 1} } \right| \le \varepsilon_{3} \hfill \\ \end{aligned}$$

where *EI*_*k*_ denotes the functional value of *EI* at the k_th_ iteration. *Y*_max_ and *Y*_min_ are the maximum and minimum responses respectively among the sample points. *f*_*k*_ and *f*_*k*-*1*_ are the values of objective functions at the *f*_*k*th_ and *f*_*k*-*1*th_ iteration respectively. $\hat{y}_{k}$ denotes the predicted value of Kriging at the *k*_th_ step. The first inequality indicates the convergence of EI. The second inequality denotes that Kriging predictive value is very close to the FEM simulated value, which means that the approximate function relationship between design objectives and design variables constructed by Kriging with high accuracy;The third inequality represents the convergence of optimization process. The execution flow chart of altered adaptive optimization method based on Kriging surrogate model is shown in Fig. 5.

## Results

### Optimization results of stent fatigue life

The design optimization of stents geometric structure were conducted to maximize the shortest distance from the data points to the failure line in order to reduce the risk of stent fatigue fracture caused by the loading of pulsation.

1. Design case of diamond-shaped stent: MRG is adopted to select 18 initial training sample points in the design space and after 17 iterations the optimization terminates.

Design parameters of stent geometry and fatigue life of the optimal stent and the original one are displayed in Table 2, from which it can be seen that the value of the design variables of *w*_1_, *w*_2_ and *t*_1_ has been reduced by 0.0116, 0.0362 and 0.0153 mm, respectively. The shortest distance from the data point to the failure line was increased by 22.39%. From GD, the stent fatigue failure may occur in the cases as follows: firstly, failure may occur during the deployment of stent by the expansion of balloon inside the stenotic artery, which involves large amounts of mean stress *σ*_*m*_. Secondly, fatigue failures may occur in the long term with a large number of pulsating loading, which defined by the amplitude of the cyclic stress *σ*_*a*_ due to the heart beating. A decrease of the width and thickness of struts results in a decrease of the radial stiffness of the stent, and then results in an increase of the amplitude of the applied cyclic stress *σ*_*a*_ and decrease of the mean of the applied stress *σ*_*m*_. Therefore, there is an optimal combination of strut width and thickness of diamond- shaped stent corresponding to the optimal fatigue life of it.

**Table 2:** Stent design optimization results

Stents	Variables (mm)			*σ*_*m*_ (MPa)	*σ*_*m*_ (MPa)	*D* ^*shortest*^ (reduced by)
	*w*_1_	*w*_2_	*t*_1_			
Diamond stent						
Original	0.28	0.249	0.12	304.62	2.02	22.39%
Optimal	0.2685	0.2128	0.1047	238.49	3.51	

Stents	Variables (mm)			*σ*_*m*_ (MPa)	*σ*_*m*_ (MPa)	*D* ^*shortest*^ (reduced by)
	*w*_1_	*R*	*t*_2_			
Sv stent						
Original	0.1	0.09	0.1	328.05	3.09	15.91%
Optimal	0.1111	0.0922	0.0838	283.48	4.78	

The shortest distance from the data point of diamond-shaped stent to the failure line was increased by 22.39%. While the shortest distance from the data point of sv-shaped stent to the failure line was increased by 22.32%

**Fig. 6:**
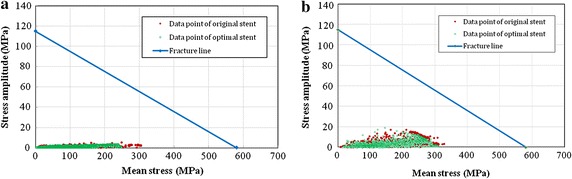
Goodman diagrams of the original and optimal stents based on diamond-shaped and sv-shaped stents, respectively. **a** *diamond*-*shaped* stent platform,**b** *sv*-*shaped* stent platform

Goodman diagrams recommended by FDA for the original and optimal diamond-shaped stents were drawn respectively as shown in Fig. 6a. In the Goodman diagrams, the points below material’s failure line are safe and the greater distance away from the fatigue limit means safer for the points under pulsation effect. After optimization, the shortest distance from the data point to the failure line was increased. It indicated that the stent becomes much more safer after optimization since the points on Goodman diagram for the optimized stent stay further from the fatigue limit.

(2) Design case of sv-shaped stent: The optimization of sv-shaped stent geometries to improve its fatigue life stopped after 10 iterations with 18 initial training sample points generated by MRG.

The optimization results were listed in Table 2. After optimization, the width of the struts was increased by 11.1%, the thickness of the stent was decreased by 7.8% and the chamfer radius was reduced by 6.2%. An increase in the width and thickness of struts results in an increase of the radial stiffness of stent, which eventually results in an decrease of amplitude of the applied stress *σ*_*a*_ and decrease of the mean of the applied stress *σ*_*m*_. Similarly,there is a optimal combination of the width and thickness of strut, as well as the chamfer radius of sv-shaped stent geometries corresponding to the optimal fatigue life of it.

Goodman diagrams of the original and the optimal stents were illustrated in Fig. 6b, in which *σ*_*a*_ is a function of *σ*_*m*_. The distance from the data point to the failure line denotes the risk of fatigue fracture of stent in service. After the structure optimization of sv-shaped stent, the shortest distance from the data point to the failure line was increased by 15.91%, which means the optimal stent has lower risk of fatigue fracture in service compared with the original design.

### Optimization results of stents expanding performance

In this part, the optimal length of balloon is searched for the optimal stent to minimize the absolute value of dogboning ratio with the aim of ensuring uniform expanding of stents and improve stents expanding performance.

1. Design case of diamond-shaped stent: MRG is employed to select 10 initial training sample points in the design space concerning the balloon’s length of diamond-shaped stent. After 5 iterations, the optimization terminated. After optimization, dogboning effect almost disappears and stent expands uniformly along its length.

Expanding performance of stent before and after the optimization of balloon’s length is compared as shown in Table 3. When the stent achieves its maximum expansion at 32 ms, stent dogboning ratio is declined completely and dogboning effect almost disappears, which means uniform expansion of optimal stent along its length. Although dogboning effect after unloading of balloon hasn’t been considered as a design objective, it is decreased by 98.16%, which implies that after unloading of balloon, dogboning effect is almost eliminated. What’s more, the proximal and distal radial elastic recoil decreases by 40.98 and 35% respectively and longitudinal recoil also declines by 1.75%. These performance indicators are related to in-stent restenosis and improvement of these indicators can reduce the occurrence of such a disease.

**Table 3:** Performance of diamond-shaped stent and sv-shaped stent deployed with original and optimal balloon

(Unit: mm)						
Stents	*L*	DR		RR		FS
		t = 32 ms	t = 42 ms	Proximal	Distal	
Diamond stent						
Original	5.1	0.0884	0.0868	0.0205	0.0220	0.2149
Optimal	4.959	0	0.0016	0.0121	0.0143	0.1974
Sv stent						
Original	6.5	0.0352	0.0363	0.0185	0.0174	0.0571
Optimal	6.0911	0	0.0027	0.0032	0.0005	0.0475

Diamond-shaped stent: (a) the dogboning effect at 32 ms is completely disappeared and dogboning ratio at 42 ms is decreased by 98.16%. The proximal and distal radial elastic recoil decreases by 40.98 and 35% respectively. Foreshortening was reduced by 1.75%. (b) sv-shaped stent: The dogboning effect at 32 ms is also completely disappeared and dogboning ratio at 42 ms is decreased by 92.56%. The proximal and distal radial elastic recoil decreases by 82.70 and 97.13% respectively. Foreshortening was reduced by 16.81%

(2) Design case of sv-shaped stent: 5 initial training samples were generated by MRG in the design space of the length of balloon placed inside of sv-shaped stent. 4 iterations were needed to obtain the optimal design. After optimization, the dogboning effect was completely eliminated.

The expansion performance of sv-shaped stent dilated by the original balloon and optimal balloon is compared as shown in Table 3. The dogboning effect of sv-shaped stent was completely eliminated after optimization, which indicates a uniform expansion along stent longitudinal direction. Similarly, as the uniform expansion is a important performance of sv-shaped stent, radial recoil at proximal and distal ends, foreshortening, as well as the dogboning ratio of stent after deflation of balloon were respectively improved by 82.70, 97.13, 16.81 and 92.56%, although they were not considered in the optimization function. The comprehensive performance of sv-shaped stent was improved after the optimization.

**Fig. 7:**
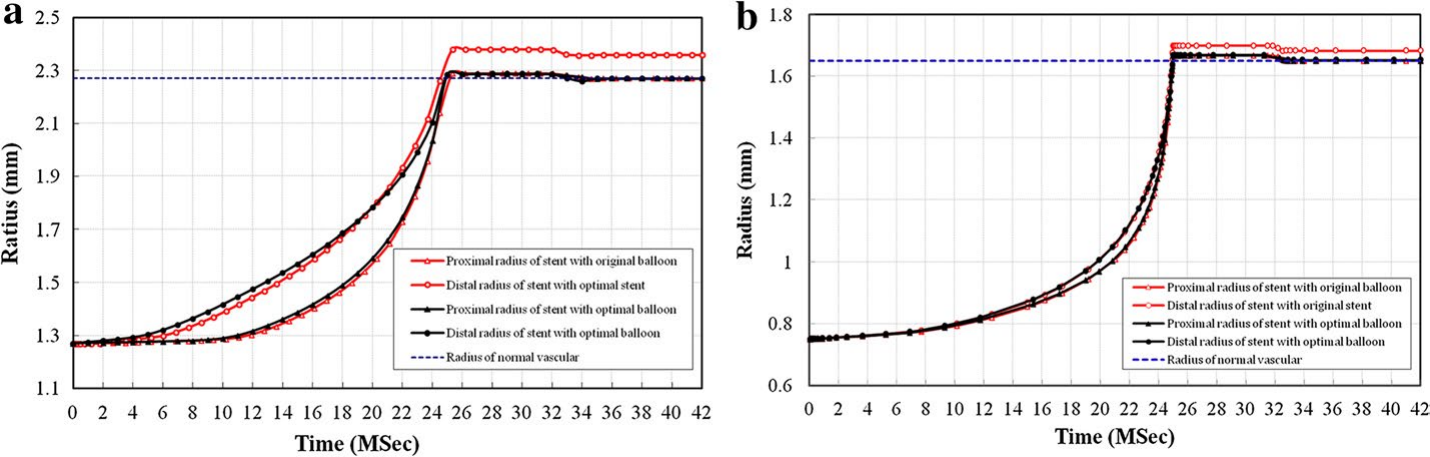
Radius of original and optimal stents during dilatation process. In first load phases 0–25 ms, both original and optimal stents were expanded gradually, but the struts didn’t reach the vessel wall until stents were fully expanded. In the second load phases 25–32 ms, the radius of the stents remained at a constant level. In the third load phases 32–42 ms, there was a small radial elastic recoil of stent, which occurred about 32–34 ms. **a** *Diamond*-*shaped* stent: the radial of normal vascular is 2.25 mm, **b** *sv*-*shaped* stent: the radial of normal vascular is 1.65 mm

The radius at the proximal and distal parts of diamond-shaped stent and sv-shaped stent, as a function of time is illustrated in Fig. 7. The period of 0–25 ms is the linear loading process during which the stent is expanded gradually and the expanding process accelerates over time and thus the difference in radius between the proximal part and the distal part of stent is relatively large. It indicates that stent’s expansion along its length is not uniform and dogboing ratio is relatively large. During the period of 25–32 ms the pressure imposed on balloon remains constant and the radiuses of stent reach the maximum and the difference in radius between the proximal and distal part of stent is relatively large due to expanding of balloon with its original length. However, the optimal balloon leads to similar radius at the proximal and distal part of stent. It shows that the optimal balloon ensures the stent to expand uniformly along its length and dogboning effect is almost eliminated. The period of 32–42 ms is unloading process during which proximal and distal radial elastic recoil occurs and the difference in radius between the proximal and distal part of stent still remains relatively large due to expanding of balloon with its original length. But the balloon with optimized length leads to similar radius at the proximal and distal part of the stent. It indicates that the optimal balloon ensures the stent to expand uniformly along its length and dogboning effect almost disappears. During the period of 25–42 ms, radiuses of stent reach the maximum and the stent contacts with vessel wall. During this time, if stent fails to expand uniformly, it would cause serious mechanical injury to vessel wall. The optimization method proposed in this study effectively avoids this damage and it is helpful to prevent ISR.

## Discussions

An optimization method based on Kriging surrogate model was adopted to optimize the stent and its expanding balloon to prolong the service life of stent and improve its expanding performance. Numerical result shows that the altered adaptive optimization method based on Kriging surrogate model can effectively optimize the stent and its expanding balloon. The black-box optimization adopting Kriging surrogate model and finite element method can not only find out the optimal result in the design space but is cheaper and more efficient than experiment and clinic test.

Whilst it is more reliable of the data from experiment, which can give a suggestion for stent design, it is hard to find the global optimal design, especially there is coupling effect between design variables. The ISAR-STEREO trials [30] provided a compelling clinical evidence for reduce restenosis with thinner struts. Nakatani et al. [31] reported that wider struts result in greater neo-intimal hyperplasia and poor stent coverage. Most of them are tend to assess one of the variables by fixing others. However, it is hard to study coupling variables, especially the Multi-objective design with coupling variables by clinical trials and experimental. Moreover, since stents are small scale devices subjected to long-term in-service loading of pulsation which is about 4 × 10^8^ cycles [32], direct experimental testing is difficult and time-consuming to perform.

Therefore, computational approaches represent an assessment tool for stent expansion performance and fatigue lifetime prediction which also considered in several regulatory bodies [3, 33]. However, the functional relationship between design parameters and design objectives of stents is nonlinear, complex, and implicit. Moreover, the multi-objective design of stents involves a number of potentially conflicting performance criteria. Most of the existing framework just studied stents performance by numerical simulation, compared the performance of different types of stents or the same type of stent with different dimensions, and provided the suggestions of stent design. It is easy to study the mechanical properties and analyze the effective factors, but it is difficult to find the globally optimal design in design space.

Therefore, finite element analysis (FEA) based computationally measurable optimization was employed for design of stent geometry. Among them, surrogate modeling methods, which predominantly involves Kriging surrogate model, was constructed to represent the relationship between design goals and design variables. Harewood et al. [34] focused on radial stiffness of stent adopting finite element analysis of a single ring. Li et al [15] optimized stent dogboning using a three-dimensional expansion model of balloon, stent, plaque and artery. Li et al [16] focused on pharmaceutically effective time of drug release in a stented artery. When considering multiple objectives, Pant et al [35] and Tammareddi et al [36] constructed and searched the Pareto fronts generated by treating each objective separately. Bressloff [4] recast the optimization as a constrained problem, wherein design improvement is sought in one objective while other objectives were considered as constraints. Among them, as a semi-parametric approach, the Kriging model is much more flexible than approaches based on parametric behavioral models.

However, a desirable stent should possess a number of excellent mechanical properties, such as (1) low metal surface coverage; (2) good flexibility; (3) enough radial strength; (4) long fatigue life; (5) low rate of longitudinal shortening; (6) low radial recoil;(7) a small amount of foreshortening; (8) small dogboning effect; (9) good expansibility; (10) good biocompatibility and so on. Therefore, multi-objective optimization of stent design involves a large number of design goals. It is difficult to find the optimal design to improve the overall performance of stenting just by one of the common methods to solve multi-objective problem, such as combining the design objectives in a single weighted objectives function, searching the Pareto fronts, executing the sub-optimizations step by step, and taking same design objectives as constraints. In future work, these methods can be used in combination under the premise of rational planning of design objectives and design variables of stent optimization systems to improve the performance of stenting. The design optimization objectives should include stent auxiliary expansion, in-stent blood flow, drug release, and biomechanical response of vascular tissue. Meanwhile, not only stent structure but also geometries of balloon, structure of polymer coating, and loading process of stent dilatation should be selected as the design variables.

In terms of optimization algorithm, accuracy of Kriging modeling relate to the distribution of simple points in the design space. Li et al [15] studied the sampling methods including Rectangle Grid (RG), Modified Rectangle Grid (MRG), Latin Hypercube Sampling (LHS), and Optimal Latin Hypercube Sampling (Optimal LHS), and pointed out that both MRG and Optimal LHS have better space-filling properties comparing to RG and LHS. Obviously, increasing the number of sample points is helpful to improving the accuracy of surrogate model. But, analysis each design on samples costs a lot of computing. Consequently, it is a challenging and opportunistic work for further systematic optimization of stenting to study better sampling strategy with a smaller number of points and more efficient surrogate modeling. Furthermore, parallel computing can be used to improve computational efficiency and save computing time.

Although computer-based method has many advantages in stent design and represents an assessment tool for stent performance prediction, it cannot completely replace the experiment studies and clinical tests. It is meaningful and challenging to bridge the gap between the engineering design optimization method and medical communities.

This study suffers from several limits such as: (a) The chemical corrosion of blood to stent hasn’t been considered when evaluating stent’s fatigue life; (b) Since stent expansion process simulation driven by cylindrical balloon has the similar results as the expansion driven by folded balloon and the simulation with cylindrical balloon can significantly save time, balloon pleating/folding hasn’t been considered during the expanding of stent; (c) The optimized results haven’t been testified by experiment and it is only an exploration of the optimization of stent and its expanding balloon.

## Conclusions

In this study, an altered adaptive optimization method based on Kriging surrogate model is proposed to optimize the stent and balloon so as to improve the fatigue life of stent as well as its expanding performance. Numerical result proves that this approach can effectively optimize the structure of stent and its expanding balloon. Multi-objective design optimization for stent and its auxiliary system shall be carried out so as to improve the overall performance of stent.

## Abbreviations

PTCA: percutaneous transluminal coronary angioplasty
ISR: in-stent restenosis
FDA: food and drug administration
FSI: fluid-structure interaction
LIPs: lagrange interpolating polynomials
ANN: artificial neural networks
MDO: multi-disciplinary design optimization
FEM: finite element method
DOE: design of experiment
MRG: modified rectangular grid
EI: expected improvement
VSMC: vascular smooth muscle cell
